# L-Theanine Regulates the Abundance of Amino Acid Transporters in Mice Duodenum and Jejunum via the mTOR Signaling Pathway

**DOI:** 10.3390/nu15010142

**Published:** 2022-12-28

**Authors:** Kehong Liu, Yingqi Peng, Ling Lin, Zhihua Gong, Wenjun Xiao, Yinhua Li

**Affiliations:** 1 Key Lab of Tea Science of Ministry of Education, Hunan Agricultural University, Changsha 410128, China; 2 National Research Center of Engineering Technology for Utilization of Botanical Functional Ingredients, Hunan Agricultural University, Changsha 410128, China; 3 Co-Innovation Center of Education Ministry for Utilization of Botanical Functional Ingredients, Changsha 410128, China; 4 Guangdong Agricultural Technology Extension Center, Guangzhou 510520, China

**Keywords:** L-theanine, amino acid transporters, mTOR signaling pathway, S6, S6K1

## Abstract

The intestine is a key organ for the absorption of amino acids. L-theanine (LTA) is a structural analog of glutamine and a characteristic non-protein amino acid found in tea (*Camellia sinensis*) that regulates lipid and protein metabolism. The present study explored the role of LTA in intestinal amino acid absorption, protein synthesis, and its mechanisms. Overall, our findings suggest that LTA supplementation not only affects serum alkaline phosphatase (AKP), total protein (TP), and urea nitrogen (BUN) levels, but it also upregulates the mRNA and protein expression of amino acid transporters (EAAT3, EAAT1, 4F2hc, y^+^LAT1, CAT1, ASCT2, and B^0^AT1), and activates the mTOR signaling pathway. The downstream S6 and S6K1 proteins are regulated, and the expression of amino acid transporters is regulated. These findings suggest that LTA increases intestinal AA absorption, promotes protein metabolism, and increases nitrogen utilization by upregulating AAT expression, activating the mTOR signaling pathway, and phosphorylating the mTOR downstream proteins S6 and S6K1.

## 1. Introduction

Amino acids (AAs) are the fundamental building blocks of protein molecules in organisms. They are closely related to the life activities of organisms and are indispensable nutrients in organisms [1]. Appropriate AA supplementation has been proven to promote health, provide essential energy, boost immunity, accelerate hormone secretion, and stimulate protein synthesis [2]. The intestinal tract is the key organ for AA absorption [3], but the small intestine cannot synthesize or store some amino acids and must obtain them from endogenous or exogenous sources [4]. Therefore, it is an urgent problem to improve the absorption capacity of AA in the small intestine and explore the possible mechanism of its influence on absorption.

Amino acids are mainly decomposed into free amino acids or small peptides by ingesting protein by pepsin, trypsin, and hydrolytic enzymes secreted by intestinal epithelial cells, or by proteases secreted by intestinal colonies, and most of them are absorbed and utilized in the intestinal tract [5,6]. Amino acids enter the portal circulation and are absorbed and utilized by extraintestinal tissues. The absorption and transport of intestinal amino acids are mediated by amino acid transporters in intestinal epithelial cells [7,8]. Factors affecting the expression of amino acid transporters can regulate the body’s absorption and transport of amino acids [9]. Animal growth and development stages, ingested nitrogen levels, growth factors, hormones, and miRNAs can all regulate the expression of amino acid transporters [10,11]. In recent years, improving intestinal absorption by ingesting plant ingredients has been considered a safer option [12]. Some plant extracts protect intestinal health by stabilizing intestinal flora and promoting the morphological development of intestinal mucosa to promote the absorption of more nutrients [13,14]. L-theanine (LTA; γ-glutamylacetamide) is a non-protein AA [15] found in tea plants (*Camellia sinensis*); its structure is similar to the γ-carboxamide group of glutamine, which can undergo N-ethylation to yield LTA. LTA regulates glucose, lipid, and protein metabolism, and it has a positive effect on the intestinal microbiota [16]. Furthermore, LTA preserves the structural integrity and morphology of the intestine and enhances nonspecific immune functions of the intestine [17]. L-theanine supplementation may be an effective way to improve the absorption of amino acids in the small intestine.

Our previous study showed that high-dose LTA (400 mg•kg^−1^•d^−1^) had a significant effect on blood ALT, TP and BUN levels, and, in addition, LTA could promote protein synthesis and improve nitrogen utilization [18]. LTA may promote protein metabolism by activating the mTOR signaling pathway. mTOR complex 1 (mTORC1) regulates protein translation, lipid and nucleotide syntheses, and autophagy. mTORC1 is regulated by many upstream signals, including nutrients, growth factors, energy, oxygen levels, and stress [19,20,21,22]. Furthermore, mTORC1 activation requires AAs such as leucine, arginine, glutamine, and serine [23,24]. Amino acid transports (AATs) initially participate in mTORC1 regulation by controlling the entry of AAs into cells [25]. Moreover, in response to the influx of specific AAs, some AATs recruit and activate mTORC1 on the surface of late endosomes and lysosomes [26,27]. Activated mTOR regulates protein translation through its downstream effector p70S6 kinase β1 (S6K1) and directly targets 4E-binding protein 1 (4E-BP1). S6K1 is a protein kinase for ribosomal protein S6; its activation is a key step in the mRNA translation of genes encoding ribosomal and some translation regulatory proteins [19].

Current research on the effect of LTA on the intestinal tract mainly focuses on protecting the intestinal tract, regulating the biological activity of the immune system, and other functions [28], but its effect on the absorption of nutrients in the gut is poorly understood. Because LTA is structurally similar to L-glutamine and has similar physiological functions, it is of great scientific and practical importance to study its effects on AA metabolism in a healthy body and determine whether it can serve as a suitable substitute for L-glutamine. Therefore, this study mainly explored the regulatory effects of LTA and L-glutamine on intestinal amino acid transporters and revealed their possible mechanisms for the first time.

## 2. Materials and Methods

### 2.1. Chemicals

LTA (purity of more than 98%) was provided by Sunfull Bio-tech Co., Ltd. (Changsha, China), and L-glutamine (LG, purity than 98%) was supplied by China Pharmaceutical Group Chemical Reagents Co., Ltd. (Shanghai, China).

Antibodies were used against B^0^AT1, y^+^LAT1, p-mTOR, p-S6K1, and p-S6 (ab180516, ab236669, ab137133, ab109393, and ab215214, respectively; Abcam, Cambridge, UK), β-actin, EAAT1, EAAT3, CAT-1, 4F2hc, mTOR, S6K1, and S6 (60008-1-Ig, 20785-1-AP, 12686-1-AP, 14195-1-AP, 15193-1-AP, 20657-1-AP, 14485-1-AP, and 14823-1-AP, respectively, Proteintech, IL, USA), and ASCT2 (5345, Cell Signaling Technology, BSN, MA, USA).

### 2.2. Experimental Design and Animals

Four-week-old male specific-pathogen-free (SPF) Kunming (KM) mice, weighing 18–22 g each were obtained from Hunan SJA Laboratory Animal Co., Ltd. (Changsha, China). The animals were housed in an environment (25 ± 2 °C) with a 12 h light/dark cycle and were allowed access to food and water ad libitum. The animals were acclimated for 3 days before the experiments were conducted. All animal procedures were performed according to the Guidelines for Animal Care and Use and the regulations of the Ethics Committee of Hunan Agriculture University (registry number: 015063506, Changsha, China).

After 3 days of adaptive rearing, 50 mice were randomly assigned to one of five treatment groups: control (CK), low dose L-theanine (LLT, 100 mg/kg/d), medium dose L-theanine (MLT, 300 mg/kg/d), high-dose L-theanine (HLT, 400 mg/kg/d), and positive control LG groups (300 mg/kg/d) (*n* = 10/group). The corresponding doses of LTA and L-glutamine in each group were dissolved in 10 mL of distilled water, and each mouse was gavaged with 0.3 mL of the solution. The normal group was fed an equal volume of distilled water (0.3 mL), and the other groups were fed the corresponding doses of LTA and L-glutamine; all mice were fed on a daily and continuous basis for 28 days. Every day, mice in each group were weighed for the remaining diet regularly, and 200 (±2) g of the diet was quantitatively added; the body weight of each mouse was weighed every other day. After the last distilled water/LTA/L-glutamine administration, all mice were starved for 12 h and euthanized using sodium pentobarbital. Blood samples (0.5 mL) were collected into heparinized tubes via the orbital sinus after the oral administration. The blood samples were centrifuged at 5071×*g* for 5 min. The small intestine was flushed with normal saline after removal. Intestinal tissues (duodenum and jejunum) and serum were collected and stored at −80 °C until analysis.

### 2.3. Detection of Blood Biochemical Indexes and Intestinal Free AAs in Mice

Serum alkaline phosphatase (AKP), alanine aminotransferase (ALT), aspartate aminotransferase (AST), urea nitrogen (BUN), glucose, albumin (Alb), total protein (TP), T-CHO, total glyceride (TG), high-density lipoprotein cholesterol (HDL-C), and low-density lipoprotein cholesterol (LDL-C) were detected at the Nanjing Jiancheng Bioengineering Institute (Nanjing, China). In brief, the intestinal samples were processed as follows: 1 mL of 0.01 mol/L hydrochloric acid was added to 100 mg of duodenum and jejunum samples and then homogenized; the mixture was diluted to 10 mL, and 2 mL of the mixture was used for detection.

An AA analyzer (Hitachi L-8900, Hitachi, Chiyoda-ku, Tokyo) was used to detect essential AAs and nonessential AAs; chromatographic conditions of amino acid analyzer: sulfonic acid cation separation column, column temperature 57 °C, reactor temperature 136 °C, mobile phase pump flow rate 0.400 mL/min, derivative pump flow rate 0.350 mL/min, and gradient elution. Essential AAs included threonine (Thr), lysine (Lys), isoleucine (Ile), leucine (Leu), valine (Val), methionine (Met), phenylalanine (Phe), tryptophan (Trp), and histidine (His). Nonessential AAs included aspartic acid (Asp), serine (Ser), glutamic acid (Glu), sarcosine (Sar), glycine (Gly), alanine (Ala), citrulline (Cit), tyrosine (Tyr), L-Ornithine (Orn), arginine (Arg), proline (Pro), cysteine (Cys), phosphoserine (P-Ser), taurine (Tau), and gamma-aminobutyric acid (GABA).

### 2.4. Reverse Transcription-Quantitative PCR

Total RNA from the duodenum and jejunum tissues was isolated using E.Z.N.A Total RNA Kit I (Omega, GA, USA) according to the manufacturer’s instructions. The isolated RNA was reverse transcribed into cDNA using the Prime Script RT Reagent Kit with gDNA Eraser (Takara-Bio, Otsu, Japan). Two-step reverse transcription-quantitative PCR (qRT-PCR) reactions were performed in an ABI QuantStudio 3 (Applied Biosystems Inc., Foster, CA, USA). Oligonucleotide primers listed in the Supplementary Table were synthesized by Sangon Bioengineering Co. Ltd. (Shanghai, China). β-Actin was used as the normalizing gene. To analyze gene expression, qRT-PCR was conducted using TB Green Premix Ex Taq II (Takara-Bio), and expression levels were calculated using the 2-(ΔΔCT) method, with the CK group as the control.

### 2.5. Western Blotting

Proteins from the duodenum and jejunum tissues were extracted using chilled RIPA buffer (Beyotime Institute of Biotechnology Inc., Haimen, China) containing a protease inhibitor cocktail (Roche Applied Science, Basel, Switzerland). The protein concentration was determined using the BCA kit (WellBio, Changsha, China). Western blotting was performed according to the method described by Gong [16].

### 2.6. Statistical Analyses

IBM SPSS 22.0 (SPSS Inc., Chicago, IL, USA) was used for data analysis. Data analysis was conducted using a one-way ANOVA with Tukey’s test to determine differences between the experimental groups. The results are expressed as mean ± SD. *p* < 0.05 was considered to indicate statistical significance.

## 3. Results

### 3.1. Effects of LTA on Serum Biochemical Indexes in Mice

To determine the therapeutic effect of LTA, we established a mouse model with LTA as the treatment group and L-glutamine as the positive control. AKP is closely related to the absorption, transport, and synthesis of proteins, carbohydrates, and fats. Serum BUN decreases when AAs and proteins are well balanced. TP reflects the absorption, transport, and metabolism of proteins. In this study, similar to the LG group, the HLT group exhibited high levels of AKP, TP, and BUN, indicating that LTA administration can enhance protein metabolism and nitrogen utilization (Table 1).

### 3.2. Effects of LTA on Free AAs in Different Intestinal Tissues of Mice

Protein metabolism is closely related to AAs, which are generally absorbed in the gut. Therefore, we examined the effect of LTA on free AAs in the gut. The results demonstrated that LTA increased the content of free AAs in the duodenum and jejunum (Table 2 and Table 3). The content of essential AAs in the duodenum (Table 2) and most of the free AAs in the jejunum (Table 3) increased with the increasing LTA dose. This finding indicates a dose-dependent effect of LTA on the content of free AAs in the intestinal tract of mice.

### 3.3. Effect of LTA on AAT mRNA Expression in Mouse Duodenum

**Figure 1:**
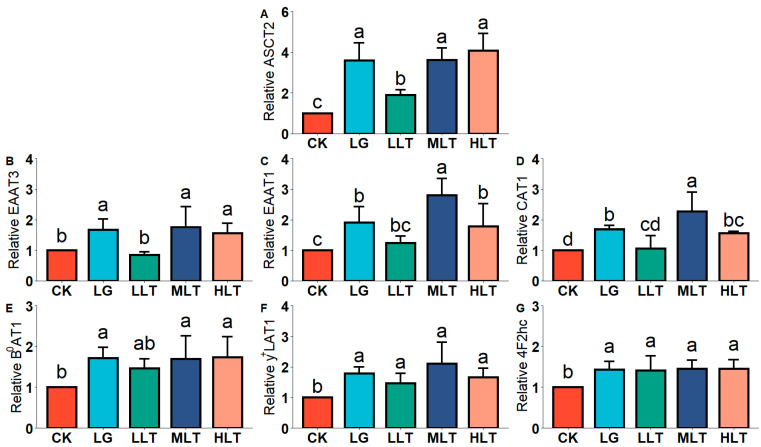
Effects of L-theanine on mRNA expression of amino acid transporters of (**A**) ASCT2, (**B**) EAAT3, (**C**) EAAT1, (**D**) CAT1, (**E**) B^0^AT1, (**F**) y^+^LAT1, and (**G**) 4F2hc in the duodenum of mice. CK (control), HLT (LTA—400 mg•kg^−1^•d^−1^), MLT (LTA—300 mg•kg^−1^•d^−1^), LLT (LTA—100 mg•kg^−1^•d^−1^), and LG (L-glutamine—300 mg•kg^−1^•d^−1^) groups. Values are presented as mean ± SD; *n* = 10/group. Different superscript letters in the same figure indicate statistically significant differences (*p* < 0.05).

To validate our results, we examined the mRNA expression of AATs to assess the effect of LTA on AATs. In the duodenum (Figure 1), compared with the CK group, except for the downregulation of EAAT3 in the LLT group (Figure 1B), ASCT2 (Figure 1A), EAAT1 (Figure 1C), CAT1 (Figure 1D), B^0^AT1 (Figure 1E), y^+^LAT1 (Figure 1F), and 4F2hc (Figure 1G) were upregulated in the LTA groups, and these levels were significantly higher in the MLT and HLT groups than in the CK group; EAAT1 and CAT1 levels in the MLT group were significantly higher than those in the LG group.

### 3.4. Effect of LTA on AAT mRNA Expression in Mouse Jejunum

**Figure 2:**
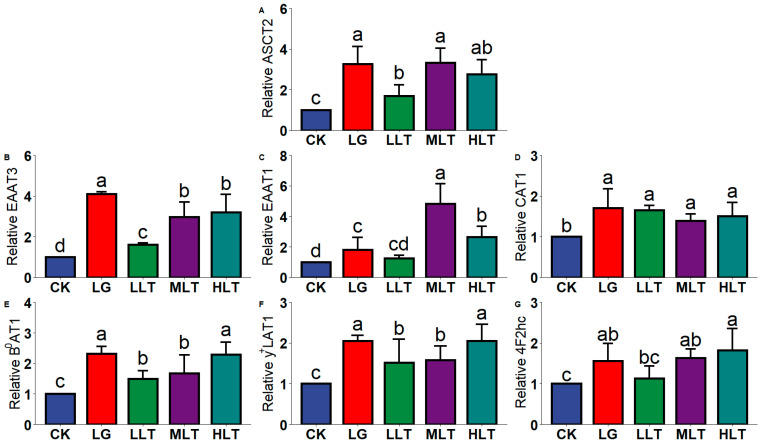
Effects of L-theanine on mRNA expression of amino acid transporters of (**A**) ASCT2, (**B**) EAAT3, (**C**) EAAT1, (**D**) CAT1, (**E**) B^0^AT1, (**F**) y^+^LAT1, and (**G**) 4F2hc in the jejunum of mice. CK (control), HLT (LTA—400 mg•kg^−1^•d^−1^), MLT (LTA—300 mg•kg^−1^•d^−1^), LLT (LTA—100 mg•kg^−1^•d^−1^), and LG (L-glutamine—300 mg•kg^−1^•d^−1^) groups. Values are presented as mean ± SD; *n* = 10/group. Different superscript letters in the same figure indicate statistically significant differences (*p* < 0.05).

We examined the mRNA expression of AATs in the jejunum (Figure 2), and the results revealed that the AAT mRNA expression of the LTA groups was significantly higher than that of the CK group. However, the AAT mRNA expression of the LLT group was lower than that of the LG group. Compared with the LG group, EAAT1 (Figure 2C) expression was significantly upregulated in the MLT and HLT groups, but EAAT3 (Figure 2B) expression was downregulated in the MLT and HLT groups (*p* < 0.05).

### 3.5. Effect of LTA on AAT Protein Expression in Mouse Duodenum

**Figure 3:**
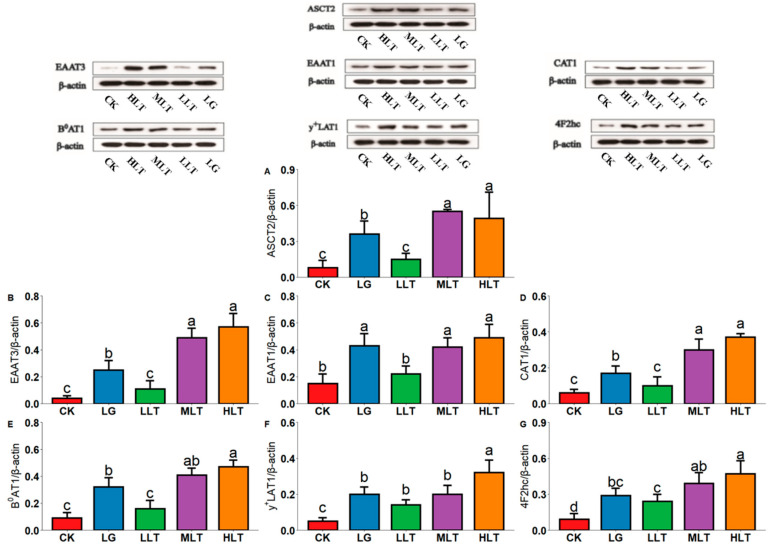
Effects of L-theanine on the protein expression of amino acid transporters of (**A**) ASCT2, (**B**) EAAT3, (**C**) EAAT1, (**D**) CAT1, (**E**) B^0^AT1, (**F**) y^+^LAT1, and (**G**) 4F2hc in the duodenum of mice. CK (control), HLT (LTA—400 mg•kg^−1^•d^−1^), MLT (LTA—300 mg•kg^−1^•d^−1^), LLT (LTA—100 mg•kg^−1^•d^−1^), and LG (L-glutamine—300 mg•kg^−1^•d^−1^) groups. Values are presented as mean ± SD; *n* = 3/group. Different superscript letters in the same figure indicate statistically significant differences (*p* < 0.05).

To further prove the reliability of our results, we detected the protein expression of related AATs. As presented in Figure 3, the expression of AAT proteins in colon tissues was significantly increased in the LTA groups. AAT protein expression was significantly higher in the HLT group than in the CK and LG groups. The protein expression of EAAT1, EAAT3, CAT1, B^0^AT1 (Figure 3B–E), and ASCT2 (Figure 3A) was significantly lower in the LLT group (*p* < 0.05) than in the LG group. By contrast, the expression of EAAT3, CAT1, and ASCT2 was significantly upregulated in the MLT group, and the expression of EAAT3 (Figure 3B), CAT1, B^0^AT1, y^+^LAT1, 4F2hc (Figure 3D–G), and ASCT2 was significantly upregulated in the HLT group (*p* < 0.05).

### 3.6. Effect of LTA on AAT Protein Expression in Mouse Jejunum

**Figure 4:**
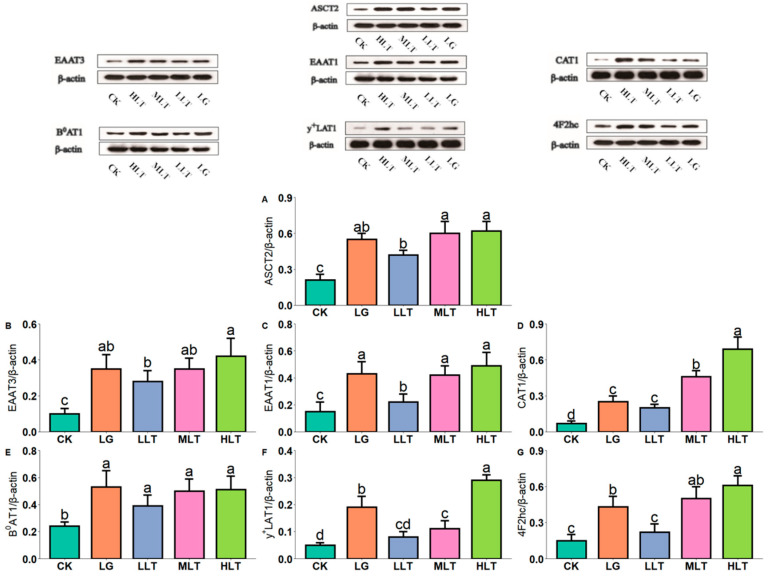
Effects of L-theanine on the protein expression of amino acid transporters of (**A**) ASCT2, (**B**) EAAT3, (**C**) EAAT1, (**D**) CAT1, (**E**) B^0^AT1, (**F**) y^+^LAT1, and (**G**) 4F2hc in the jejunum of mice. CK (control), HLT (LTA—400 mg•kg^−1^•d^−1^), MLT (LTA—300 mg•kg^−1^•d^−1^), LLT (LTA—100 mg•kg^−1^•d^−1^), and LG (L-glutamine—300 mg•kg^−1^•d^−1^) groups. Values are presented as mean ± SD; *n* = 3/group. Different superscript letters in the same figure indicate statistically significant differences (*p* < 0.05).

Figure 4 presents AAT protein expression in the mouse jejunum. In this study, compared with the CK group, the expression of AATs in the jejunum was significantly increased in the LTA groups. Furthermore, AAT expression in the LG group was significantly lower than that in the HLT group, except for EATT1 (Figure 4C).

### 3.7. Effects of LTA on mTOR, S6K1, and S6 Phosphorylation Levels

**Figure 5:**
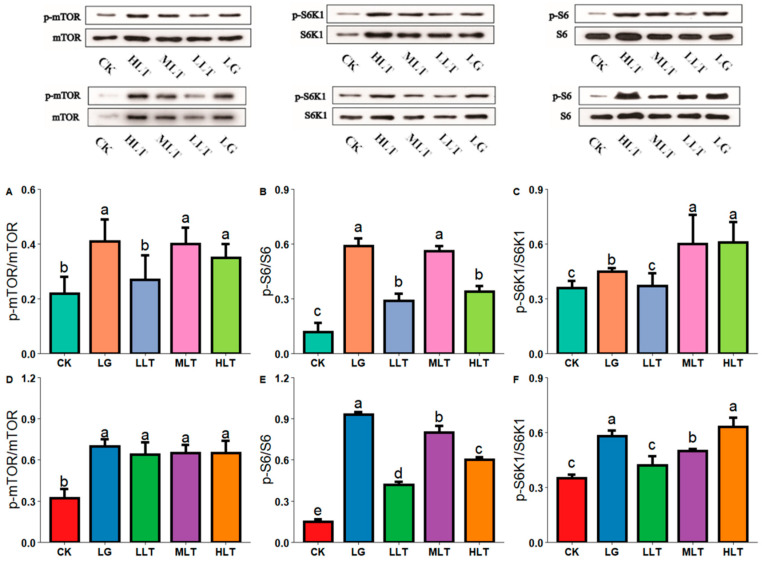
Effects of L-theanine on the phosphorylation status of proteins of mTOR, S6K1, and S6 in the jejunum (**A**–**C**) and duodenum (**D**–**F**) of mice. CK (control), HLT (LTA—400 mg•kg^−1^•d^−1^), MLT (LTA—300 mg•kg^−1^•d^−1^), LLT (LTA—100 mg•kg^−1^•d^−1^), and LG (L-glutamine—300 mg•kg^−1^•d^−1^) groups. Values are presented as mean ± SD; *n* = 3/group. Different superscript letters in the same figure indicate statistically significant differences (*p* < 0.05).

AAs can affect the expression of AATs by regulating the mTOR signaling pathway [29]. Therefore, the mTOR signaling pathway and the phosphorylation level of downstream S6K1 and S6 proteins were determined in this study. Among all treatment groups, the MLT and HLT groups exhibited the highest level of S6K1 phosphorylation in the jejunum (Figure 5A–C). Compared with the CK and LG groups, the HLT group exhibited higher levels of S6K1 phosphorylation in the duodenum (Figure 5D–F).

## 4. Discussion

Our results showed that the medium and high doses of L-theanine and L-glutamine significantly increased the expression of amino acid transporter proteins in the intestinal tissues of mice. The study shows that 100~500 mg/kg theanine is the commonly used concentration in the mouse model. In our study, the medium and high-dose concentrations were 300 and 400 mg/kg respectively. The daily dose converted from mice to humans is 1440~1920 mg per day; It is considered safe for adults to take 840 mg L-theanine every day for 4 weeks, so the cumulative dose of L-theanine is (840 mg/day × 7 days × 4 weeks = 23.52 g) higher than the inferred dose in our study (1920 mg/day × 7 days = 13.44 g), therefore, we believe that it is reasonable to give the mice 300 and 400 mg/kg of theanine for only 7 days [30,31,32,33]. Arg, Gly, Glu, and Orn are essential AAs for maintaining a healthy and functioning gut [34]. In the present study, LTA treatment increased the content of these essential AAs. LTA is beneficial for the absorption of AAs by intestinal epithelial cells for protein synthesis and cell proliferation. Trp, Gly, Met, His, and Ser are one-carbon AAs that can be used as raw materials for the synthesis of purine and pyrimidine, and they play an important role in the metabolism of nucleic acid and nucleotides. The expression of Trp, Gly, Met, His, and Ser in the intestinal tract was significantly increased in the LTA groups, indicating that LTA may promote intestinal cell growth by affecting the synthesis of DNA and RNA [35]. Furthermore, no significant difference was observed in feed intake among the treatment groups (Figure S1). BUN levels decreased in the MLT and HLT groups, and the contents of AKP and TP increased, indicating that LTA improved the ratio of AAs absorbed by the body and increased the concentration of AAs in the body, thereby promoting protein synthesis in the body. At the same, an administered dose (300 mg/kg/d) of LTA increased Pro, Asp, Glu, Ala, and Ser levels, and lowered Orn, Arg, Lys, and His levels in the duodenum compared to LG. While in the jejunum compared to LG, LTA improved the levels of most of the amino acids such as Orn, Arg, and Lys. Combining the contents of both intestinal amino acids, LTA at the same dose showed a superior effect in improving intestinal amino acid absorption than LG.

To investigate the mechanism of LTA affecting intestinal amino acid absorption, we further investigated the effect of intestinal amino acid transporter proteins ASCT2, EAAT3, EAAT1, etc. The results showed that the expression of most amino acid transport proteins was significantly increased by LTA and LG at the same dose, while the expression of ASCT2, EAAT3, and CAT1 was significantly higher by LTA than by LG, suggesting that LTA may be more effective in enhancing amino acid absorption in the intestine by increasing the expression of amino acid transport proteins. AATs activate AA absorption on sensing AA signals. This study demonstrated that LTA and LG significantly upregulated (*p* < 0.05) the mRNA and protein expression of B^0^AT1, ASCT2, 4F2hc, y^+^LAT1, EAAT3, EAAT1, and CAT1 in the small intestine of mice. A previous study reported that LTA promoted the growth of broilers by increasing the mRNA levels of intestinal AA and peptide transporters [36]. Our results are partially consistent with our previous findings; we had previously reported that LTA and LG increase the concentration of free AAs in the duodenum and jejunum of mice, indicating that they have a significant effect on AA intestinal absorption through AATs. Both compounds exhibit similar effects likely due to their structural similarity. Furthermore, the amount and source of dietary AAs have been reported to regulate the expression of cationic AATs in pigs [37]. This present study demonstrated that the mRNA and protein expression levels of most AATs in the MLT and HLT groups were significantly higher than those in the LLT group. This may be related to the increase in intracellular and extracellular AA concentrations following the administration of various LTA doses, indicating that LTA can directly affect the expression of AAT proteins. This conclusion was confirmed by the results of the correlation analysis between the LTA concentration and AATs, and the expression of AAT proteins was significantly and positively correlated with the LTA concentration (Figure S2).

Furthermore, our previous study demonstrated that in the HLT group, LTA (400 mg•kg^−1^•d^−1^) had a significant effect on blood ALT, TP, and BUN levels, similar to the effects in the LG group; therefore, both LTA and LG can promote protein synthesis and increase the nitrogen utilization rate. Approximately 9–12% of proteins are synthesized in the small intestine in animals [38], and this is closely related to their nutrient intake level [39]. Therefore, LTA may promote protein metabolism by activating the mTOR signaling pathway. The higher increase in the phosphorylation levels of mTOR, S6K1, and S6 in the MLT and HLT groups than that in the LLT group may be attributed to the effect of the extracellular AA concentration on the mTOR signaling pathway. Studies have reported that LTA increased the phosphorylation levels of the mTOR protein and its downstream proteins S6K1 and S6, as well as upregulated the expression of the neutral AAT SNAT1 (*SLC38A1*) in undifferentiated neural cells [40]. According to the results of our correlation analysis, LTA may not directly affect the expression of AAT proteins by activating the mTOR pathway but may rather phosphorylate the downstream S6K1 protein after activating the mTOR pathway, thereby affecting the expression of AAT proteins. The mTOR signaling pathway is closely related to life activities, and the mechanisms through which AAs activate mTOR are different. The activation of the mTOR pathway by glutamine requires v-ATPase and lysosomes. LTA can compete with glutamine as a carrier, causing changes in the concentration of glutamine within cells, thereby achieving neuroprotective and antidepressant effects [41]. LTA increases the phosphorylation level of downstream proteins in the mTOR signaling pathway, which may be related to changes in the concentration of intracellular glutamine. Although LTA is an analog of glutamine and has the same effect on mTOR signaling pathway-related proteins and glutamine in the intestinal tract, it is unclear how LTA regulates the mTOR signaling pathway to promote the expression of AATs. The effect remains to be studied in depth.

In this study, although LTA and LG had the same effect on intestinal AAs, LTA decreased the content of serum free AAs, whereas LG had the opposite effect. We evaluated the protein expression of AATs and the mTOR signaling pathway to determine whether the different effects of LTA and LG resulted in variations in intestinal AA absorption and protein synthesis. We speculated that because glutamine and LTA have different catabolic sites and physiological functions, their effects on AA metabolism would differ. As the glutamine utilization rate increased, the consumption of other AAs in the jejunum and ileum decreased [42]. Approximately 55% of the total glutamine intake is absorbed in the intestine [43]. The glutamine acylamino and amino groups serve as a nitrogen source for the synthesis of several important biological molecules (i.e., nonessential AAs, nucleotides, and hexosamine) to continually fuel cell proliferation [44]. LTA is absorbed in the intestine and hydrolyzed to glutamic acid and ethylamine in the kidneys [45]. Unlike glutamine, glutamic acid in the arterial blood is rarely absorbed and utilized by the small intestine, suggesting that LTA does not provide energy for small intestine processes. Furthermore, the structure of an organism is complex, and maintaining systematic AA homeostasis requires differentiation and coordination among various organs; these processes include intestinal absorption, renal reabsorption, biosynthesis, and degradation of AAs and proteins in all organs, which are under central nervous system and hormone regulation based on protein and AA intake and metabolism [46]. Although glutamine and LTA can regulate the distribution of free AAs in the intestine, the differences between their metabolic effects on AAs in the muscles, liver, and even the entire body require further investigation. Nevertheless, LTA has been reported to inhibit glutamate transporters in tumors through the GS-X pump [47]. Additionally, metabolites regulate many essential cellular activities, such as cell signaling, energy transfer, and intercellular communication [48]. Therefore, metabolomics and molecular biology techniques can be used to identify the various endogenous metabolites that may cause changes in AA metabolism in mice, elucidate the metabolic pathway network, and systematically reveal the molecular mechanism through which LTA affects AA metabolism [49]. A comprehensive comparison between the effects of LTA and glutamine on metabolomics may reveal the reasons for their differing effects on AA and protein metabolism. Although LTA is a glutamine analog and has the same effect on mTOR signaling pathway-related proteins as glutamine in the gut, how LTA modulates the mTOR signaling pathway and its effects on other amino acid transporters is the next-step focus of inquiry.

## 5. Conclusions

We demonstrated that both LTA and glutamine increase the intestinal free AA content by upregulating the mRNA and protein expression of AATs (EAAT3, EAAT1, 4F2hc, y^+^LAT1, CAT1, ASCT2, and B^0^AT1) and increase the phosphorylation levels of mTOR, S6K1, and S6 in the duodenum and jejunum of mice. These findings suggest that LTA increases intestinal AA absorption, promotes protein metabolism, and increases nitrogen utilization by upregulating AAT expression, activating the mTOR signaling pathway, and phosphorylating the mTOR downstream proteins S6 and S6K1.

## Figures and Tables

**Table 1:** Effects of L-theanine on serum biochemical indexes in mice. CK (control), HLT (LTA—400 mg•kg^−1^•d^−1^), MLT (LTA—300 mg•kg^−1^•d^−1^), LLT (LTA—100 mg•kg^−1^•d^−1^), and LG (L-glutamine—300 mg•kg^−1^•d^−1^) groups. Different superscript letters in the same table indicate statistically significant differences.

	CK	LG	LLT	MLT	HLT
TP/(g/L)	56.69 ± 11.29 ^a^	71.37 ± 19.39 ^b^	54.34 ± 8.54 ^ab^	59.49 ± 17.21 ^ab^	76.98 ± 18.26 ^b^
TG/(mmol/L)	1.39 ± 0.44 ^ab^	1. 86 ± 0.61 ^c^	1.06 ± 0.31 ^a^	1.34 ± 0.21 ^ab^	1.74 ± 0.69 ^bc^
AST/(U/L)	101.24 ± 18.98	106.23 ± 11.84	110.2 ± 24.56	100.41 ± 35.56	110.21 ± 26.31
ALT/(U/L)	34.17 ± 2.98	35.99 ± 5.74	35.28 ± 5.42	37.89 ± 6.01	36.25 ± 6.33
LDL-C/(mmol/L)	1.62 ± 0.81	1.57 ± 0.63	1.51 ± 0.48	1.66 ± 0.71	1.59 ± 0.84
Alb/(g/L)	38.59 ± 3.46	40.09 ± 8.33	35.39 ± 5.08	42.33 ± 8.21	41.84 ± 9.34
BUN/(mmol/L)	7.62 ± 0.54 ^b^	6.79 ± 0.59 ^a^	7.12 ± 0.26 ^b^	7.02 ± 0.21 ^a^	7.47 ± 0.26 ^a^
Glucose/(mmol/L)	4.39 ± 0.81	5.12 ± 1.82	4.55 ± 1.26	5.39 ± 0.95	4.96 ± 1.38
T-CHO/(mmol/L)	4.33 ± 0.81	4.32 ± 0.58	4.06 ± 0.58	4.87 ± 0.39	4.22 ± 0.79
HDL-C/(mmol/L)	2.04 ± 0.29	2.39 ± 0.34	1.77 ± 0.24	2.06 ± 0.74	2.1 ± 0.63
AKP/(U/L)	158.51 ± 24.98 ^a^	171.58 ± 16.68 ^b^	137.28 ± 11.25 ^a^	151.34 ± 12.39 ^a^	161.24 ± 24.33 ^b^

**Table 2:** Effects of L-theanine on amino acid content in mouse duodenum. CK (control), HLT (LTA—400 mg•kg^−1^•d^−1^), MLT (LTA—300 mg•kg^−1^•d^−1^), LLT (LTA—100 mg•kg^−1^•d^−1^), and LG (L-glutamine—300 mg•kg^−1^•d^−1^) groups. Different superscript letters in the same table indicate statistically significant differences

	CK	LG	LLT	MLT	HLT	Transporter
Orn	159.97 ± 23.14 ^a^	362.35 ±25.85 ^c^	276 ± 22.36 ^b^	255.84 ± 23.28 ^b^	256.32 ± 15.32 ^b^	CAT1
Arg	1458.78 ± 158.89 ^a^	2175.23 ±76.39 ^c^	1489.36 ± 124.27 ^a^	1839.42 ± 310.22 ^b^	2043.5 ± 168.58 ^bc^	CAT1, 4F2hc+y^+^LAT1
Lys	1192.08 ± 198.74 ^a^	2038.21 ± 144.43 ^d^	1570.75 ±126.37 ^b^	1748.23 ±188.96 ^c^	1885.23 ± 126.66 ^cd^	CAT1, 4F2hc+y^+^LAT1
His	240.13 ± 24.36 ^a^	341.25 ± 23.79 ^bc^	332.26 ± 17.32 ^b^	358.28 ± 15.32 ^bc^	355.56 ±16.2 ^c^	CAT1, 4F2hc+y^+^LAT1
Gly	651.14 ± 15.87 ^a^	746.36 ± 28.97 ^b^	842.89 ± 34.82 ^c^	903.25 ± 34.45 ^d^	855.23 ± 56.32 ^c^	B^0^AT1
Ile	683.31 ± 43.28 ^a^	1004.3 ±58.89 ^b^	921.57 ± 48.59 ^b^	1005.37 ± 44.45 ^b^	1084.23 ± 48.96 ^c^	B^0^AT1
Val	632.79 ± 58.23 ^a^	894.28 ± 98.74 ^bc^	826.52 ± 83.59 ^b^	966.2 ± 87.32 ^bc^	967.78 ± 98.56 ^c^	B^0^AT1
Phe	913.17 ± 88.23 ^a^	1527.18 ± 148.58 ^c^	1189.37 ± 86.59 ^b^	1244.59 ± 245.6 ^bc^	1396 ± 133.58 ^c^	B^0^AT1
Tyr	873.56 ± 94.31 ^a^	1501.2 ± 103.25 ^c^	1221.35 ± 122.32 ^b^	1127 ± 213.31 ^bc^	1298.98 ± 188.65 ^c^	B^0^AT1
Trp	264.85 ± 23.37 ^a^	415.01 ± 43.23 ^cd^	322.58 ± 34.32 ^b^	401.12 ± 48.86 ^bc^	387.86 ± 43.23 ^c^	B^0^AT1
Pro	473.85 ± 76.36 ^a^	573.18 ± 96.26 ^ab^	538.28 ± 43.54 ^a^	711.9 ± 97.23 ^c^	654.59 ± 84.23 ^bc^	B^0^AT1
Asp	769.48 ± 39.28 ^a^	845.58 ± 23.36 ^ab^	911.23 ± 41.25 ^b^	1211.23 ± 56.59 ^c^	943.82 ± 58.64 ^b^	EAAT1, EAAT3
Glu	1628.12 ± 141.28 ^a^	1889 ± 58.72 ^b^	1844.28 ± 87.27 ^b^	2335.2 ± 156.36 ^c^	2021.32 ± 144.23 ^b^	EAAT1, EAAT3
Ala	1003.25 ± 48.69 ^a^	1012.29 ± 56.34 ^c^	1186.58 ± 46.69 ^b^	1354.23 ± 54.39 ^d^	1417.68 ± 18.37 ^d^	ASCT2, B^0^AT1
Ser	799.31 ± 36.52 ^a^	965.33 ± 28.34 ^b^	1000.23 ± 33.28 ^b^	1133.57 ± 134.34 ^c^	1078.23 ± 62.58 ^c^	ASCT2, B^0^AT1
Cys	14.88 ± 2.02 ^a^	21.15 ± 2.18 ^c^	18.23 ± 1.3 ^b^	21.64 ± 2.37 ^c^	22.89 ± 5.35 ^c^	ASCT2, B^0^AT1
Thr	591.48 ± 54.28 ^a^	803.26 ± 69.56 ^bc^	784.56 ± 72.23 ^b^	831.26 ± 58.88 ^c^	852.69 ± 48.83 ^c^	ASCT2, B^0^AT1
Met	229.14 ± 20.37 ^a^	358 ± 28.35 ^c^	288.37 ± 35.28 ^b^	343.27 ± 48.29 ^bc^	348.33 ± 33.23 ^c^	4F2hc+y^+^LAT1, B^0^AT1
Leu	1382.85 ± 93.21 ^a^	2315.28 ± 232.28 ^c^	2002.31 ± 245.58 ^b^	2012.67 ± 94.73 ^b^	2338 ± 83.26 ^c^	4F2hc+y^+^LAT1, B^0^AT1

**Table 3:** Effects of L-theanine on amino acid content in mouse jejunum. CK (control), HLT (LTA—400 mg•kg^−1^•d^−1^), MLT (LTA—300 mg•kg^−1^•d^−1^), LLT (LTA—100 mg•kg^−1^•d^−1^), and LG (L-glutamine—300 mg•kg^−1^•d^−1^) groups. Different superscript letters in the same table indicate statistically significant differences

	CK	LG	LLT	MLT	HLT	Transporter
Orn	107.31 ± 8.01 ^a^	131.24 ± 12.1 ^b^	135.3 ± 21.09 ^b^	171.85 ± 21.33 ^c^	213.25 ± 42.32 ^d^	CAT1
Arg	1389.69 ± 184.43 ^a^	1784.38 ± 201.32 ^b^	1398.21 ± 84.67 ^a^	2258.33 ± 142.25 ^c^	2731.78 ± 200.8 ^d^	CAT1, 4F2hc+y^+^LAT1
Lys	1000.74 ± 135.32 ^a^	1321.54 ± 142.32 ^b^	1352.3 ± 162.36 ^b^	1821.5 ± 274.36 ^c^	2001.46 ± 312.25 ^d^	CAT1, 4F2hc+y^+^LAT1
His	274.32 ± 30.26 ^a^	371.16 ± 31.28 ^b^	263.54 ± 23.14 ^a^	374.18 ± 51.39 ^b^	513.45 ± 105.11 ^c^	CAT1, 4F2hc+y^+^LAT1
Gly	1471.41 ± 103.54 ^a^	1698.87 ± 103.9 ^a^	1691.34 ± 203.1 ^a^	2256.64 ± 266.32 ^b^	2301.32 ± 288.87 ^b^	B^0^AT1
Ile	864.59 ± 144.36 ^a^	1031.48 ± 85.38 ^b^	1024.43 ± 214.36 ^b^	1632.12 ± 103.74 ^c^	1701.25 ± 587.32 ^c^	B^0^AT1
Val	846.34 ± 113.58 ^a^	1058.64 ± 148.32 ^b^	1034.25 ± 132.71 ^ab^	15385.37 ± 233.41 ^c^	1721.82 ± 186.54 ^d^	B^0^AT1
Phe	1161.47 ± 153.73 ^a^	1284.3 ± 201.31 ^a^	1187.6 ± 177.36 ^a^	1864.64 ± 135.37 ^b^	2121.53 ± 155.31 ^c^	B^0^AT1
Tyr	1196.54 ± 105.41 ^a^	1498.29 ± 102.3 ^b^	1289.54 ± 86.52 ^a^	2013.54 ± 143.54 ^c^	2418.31 ± 112.35 ^d^	B^0^AT1
Trp	322.3 ± 53.2 ^a^	379.27 ± 52.54 ^b^	339.73 ± 27.64 ^ab^	499.28 ± 29.34 ^c^	562.27 ± 56.38 ^c^	B^0^AT1
Pro	341.58 ± 27.61 ^a^	446.29 ± 56.35 ^b^	584.6 ± 69.45 ^c^	801.38 ± 84.65 ^d^	881.83 ± 74.39 ^d^	B^0^AT1
Asp	1337 ± 172.47 ^a^	1293.4 ± 112.3 ^a^	1487.25 ± 201.36 ^a^	1881.26 ± 136.97 ^b^	1843.21 ± 111.36 ^b^	EAAT1, EAAT3
Glu	3986.3 ± 397.84 ^a^	4026.76 ±503.29 ^a^	4258.31 ±871.24 ^ab^	5084.63 ±614.85 ^b^	5023.65 ± 540.32 ^b^	EAAT1, EAAT3
Ala	2010.48 ± 121.59 ^a^	2085.64 ± 103.2 ^a^	2489.66 ± 187.36 ^b^	2917.35 ± 147.23 ^c^	3128.25 ± 188.98 ^c^	ASCT2, B^0^AT1
Ser	1001.54 ± 82.38 ^a^	1336.57 ± 142.05 ^b^	1022.59 ± 124.31 ^a^	1501 ± 142.32 ^c^	1854.58 ± 155.32 ^d^	ASCT2, B^0^AT1
Cys	25.84 ± 1.89 ^a^	30.28 ± 2.56 ^b^	28.56 ± 2.33 ^ab^	31.08 ± 3.56 ^b^	30.02 ± 3.21 ^b^	ASCT2, B^0^AT1
Thr	784.25 ± 74.66 ^a^	978.24 ± 143.69 ^b^	996.27 ± 100.2 ^b^	1321.28 ± 91.23 ^c^	1563.21 ± 108.28 ^d^	ASCT2, B^0^AT1
Met	271.69 ± 26.31 ^a^	361.32 ± 62.38 ^b^	321.83 ± 41.85 ^ab^	459.26 ± 71.23 ^c^	504.1 ± 58.23 ^c^	4F2hc+y^+^LAT1, B^0^AT1
Leu	1842.36 ± 351.87 ^a^	2018.37 ± 312.56 ^a^	2063.89 ± 141.54 ^a^	3233.16 ± 399.98 ^b^	3269.58 ± 532.34 ^b^	4F2hc+y^+^LAT1, B^0^AT1

## Data Availability

Not applicable.

## Supplementary Materials

The following supporting information can be downloaded at: https://www.mdpi.com/article/10.3390/nu15010142/s1, Figure S1. Effects of L-theanine on (A) weight and (B) feed intake of mice. Figure S2. Correlation between the AATs protein expression and n(LTA), mTOR, S6K1, and S6.

## References

[1] Li M., Li Q., Zheng Y., Shi X., Zhang J., Chuang M., Guan B., Peng Y., Yang M., Yue X. (2020). New insights into the alterations of full spectrum amino acids in human colostrum and mature milk between different domains based on metabolomics. Eur. Food Res. Technol..

[2] Bifari F., Ruocco C., Decimo I., Fumagalli G., Valerio A., Nisoli E. (2017). Amino acid supplements and metabolic health: A potential interplay between intestinal microbiota and systems control. Genes Nutr..

[3] Wu G. (2019). Amino acids: Metabolism, functions, and nutrition. Amino Acids.

[4] Liu A., Gong Z., Lin L., Xu W., Zhang T., Zhang S., Li Y., Chen J., Xiao W. (2019). Effects of L-theanine on glutamine metabolism in enterotoxigenic Escherichia coli (E44813)-stressed and non-stressed rats. J. Funct. Foods.

[5] Tian T., Wang Z., Zhang J. (2017). Pathomechanisms of Oxidative Stress in Inflammatory Bowel Disease and Potential Antioxidant Therapies. Oxid. Med. Cell. Longev..

[6] Rawla P., Barsouk A. (2019). Epidemiology of gastric cancer: Global trends, risk factors and prevention. Prz. Gastroenterol..

[7] Oliveira E.D., Burini R.C., Jeukendrup A. (2014). Gastrointestinal complaints during exercise: Prevalence, etiology, and nutritional recommendations. Sports Med..

[8] Jeppesen P.B. (2014). Spectrum of short bowel syndrome in adults: Intestinal insufficiency to intestinal failure. JPEN J. Parenter. Enteral. Nutr..

[9] Camargo S.M., Vuille-dit-Bille R.N., Meier C.F., Verrey F. (2020). ACE2 and gut amino acid transport. Clin. Sci..

[10] Jochems P.G., Garssen J., Van Keulen A.M., Masereeuw R., Jeurink P.V. (2018). Evaluating human intestinal cell lines for studying dietary protein absorption. Nutrients.

[11] Odriozola L., Corrales F.J. (2015). Discovery of nutritional biomarkers: Future directions based on omics technologies. Int. J. Food Sci. Nutr..

[12] Grigore A., Cord D., Tanase C., Albulescu R. (2020). Herbal medicine, a reliable support in COVID therapy. J. Immunoass. Immunochem..

[13] Michiels J., Missotten J., Van Hoorick A., Ovyn A., Fremaut D., De Smet S., Dierick N. (2010). Effects of dose and formulation of carvacrol and thymol on bacteria and some functional traits of the gut in piglets after weaning. Arch. Anim. Nutr..

[14] Liu Y., Song M., Che T.M., Lee J.J., Bravo D., Maddox C.W., Pettigrew J.E. (2014). Dietary plant extracts modulate gene expression profiles in ileal mucosa of weaned pigs after an Escherichia coli infection. J. Anim. Sci..

[15] Juneja L.R., Chu D.C., Okubo T., Nagato Y., Yokogoshi H. (1999). L-theanine—A unique amino acid of green tea and its relaxation effect in humans. Trends Food Sci. Technol..

[16] Lin L., Zeng L., Liu A., Peng Y., Yuan D., Zhang S., Li Y., Chen J., Xiao W., Gong Z. (2020). L-theanine regulates glucose, lipid, and protein metabolism via insulin and AMP-activated protein kinase signaling pathways. Food Funct..

[17] Gong Z., Lin L., Liu Z., Zhang S., Liu A., Chen L., Liu Q., Deng Y., Xiao W. (2019). Immune-modulatory effects and mechanism of action of l-theanine on etec-induced immune-stressed mice via nucleotide-binding oligomerization domain-like receptor signaling pathway. J. Funct. Foods.

[18] Li C., Tong H., Yan Q., Tang S., Han X., Xiao W., Tan Z. (2016). L-Theanine improves immunity by altering TH2/TH1 cytokine balance, brain neurotransmitters and expression of phospholipase C in rat hearts. Med. Sci. Monit..

[19] Saxton R.A., Sabatini D.M. (2017). mTOR signaling in growth, metabolism, and disease. Cell.

[20] Gao X., Zhang Y., Arrazola P., Hino O., Kobayashi T., Yeung R.S., Pan D. (2002). Tsc tumour suppressor proteins antagonize amino-acid–TOR signalling. Nat. Cell. Biol..

[21] Inoki K., Zhu T., Guan K.L. (2003). TSC2 mediates cellular energy response to control cell growth and survival. Cell.

[22] Sengupta S., Peterson T.R., Sabatini D.M. (2010). Regulation of the mTOR complex 1 pathway by nutrients, growth factors, and stress. Mol. Cell..

[23] Jewell J.L., Kim Y.C., Russell R.C., Yu F.X., Park H.W., Plouffe S.W., Tagliabraccl V.S., Guan K.L. (2015). Differential regulation of mTORC1 by leucine and glutamine. Science.

[24] Wang X., Campbell L.E., Miller C.M. (1998). Amino acid availability regulates p70 S6 kinase and multiple translation factors. Biochem. J..

[25] Beugnet A., Tee A.R., Taylor P.M. (2003). Regulation of targets of mTOR (mammalian target of rapamycin) signalling by intracellular amino acid availability. Biochem. J..

[26] Wyant G.A., Abu-Remaileh M., Wolfson R.L., Chen W.W., Freinkman E., Danai L.V., Mattew G., Heiden V., Sabatini D.M. (2017). mTORC1 activator SLC38A9 is required to efflux essential amino acids from lysosomes and use protein as a nutrient. Cell.

[27] Goberdhan D.C. (2010). Intracellular amino acid sensing and mTORC1-regulated growth: New ways to block an old target?. Curr. Opin. Investig. Drugs..

[28] Wang D., Cai M., Wang T., Liu T., Huang J., Wang Y., Granato D. (2020). Ameliorative effects of L-theanine on dextran sulfate sodium induced colitis in C57BL/6J mice are associated with the inhibition of inflammatory responses and attenuation of intestinal barrier disruption. Food Res. Int..

[29] Guo M., Li N., Zheng J., Wang W., Wu J. (2021). Epigenetic regulation of hepatocellular carcinoma progression through the mtor signaling pathway. Can. J. Gastroenterol. Hepatol..

[30] Kandasamy P., Zlobec I., Nydegger D.T., Pujol-Giménez J., Bhardwaj R., Shirasawa S., Tsunoda S., Hediger M.A. (2021). Oncogenic KRAS mutations enhance amino acid uptake by colorectal cancer cells via the hippo signaling effector YAP1. Mol. Oncol..

[31] Miho O., Chisato W., Noriko S., Hiroaki H., Kotaro H., Toshita T., Hayato O., Tsutomu O., Hiroshi K. (2015). Effffect of L-theanine on glutamatergic function in patients with schizophrenia. Acta Neuropsychiatr..

[32] Sarris J., Byrne G.J., Cribb L., Oliver G., Murphy J., Macdonald P. (2019). L-theanine in the adjunctive treatment of generalized anxiety disorder: A double-blind, randomised, placebo-controlled trial. J. Psychiatr. Res..

[33] Tsuchiya T., Honda H., Oikawa M., Kakita T., Oyama A., Oishi H., Tochikubo K., Hashimoto T., Kurihara S., Shibakusa T. (2016). Oral administration of the amino acids cystine and theanine attenuates the adverse events of S-1 adjuvant chemotherapy in gastrointestinal cancer patients. Int. J. Clin. Oncol..

[34] Ma N., Ma X. (2019). Dietary amino acids and the gut-microbiome-immune axis: Physiological metabolism and therapeutic prospects. Compr. Rev. Food Sci. Food Saf..

[35] Zhang L., Ma M., Li Z., Zhang H., He X., Song Z. (2021). Protective Effects of L-Theanine on IPEC-J2 Cells Growth Inhibition Induced by Dextran Sulfate Sodium via p53 Signaling Pathway. Molecules.

[36] Zhang C., Wang C., Chen K., Zhao X., Geng Z. (2020). Effect of L-theanine on growth performance, intestinal development and health, and peptide and amino acid transporters expression of broilers. J. Sci. Food Agric..

[37] García-Villalobos H., Morales-Trejo A., Araiza-Piña B.A., Htoo J.K., Cervantes-Ramírez M. (2012). Effects of dietary protein and amino acid levels on the expression of selected cationic amino acid transporters and serum amino acid concentration in growing pigs. Arch. Anim. Nutr..

[38] Simon O., Bergner H., Münchmeyer R., Zebrowska T. (1982). Studies on the range of tissue protein synthesis in pigs: The effect of thyroid hormones. Br. J. Nutr..

[39] Stoll B., Chang X., Fan M.Z., Reeds P.J., Burrin D.G. (2000). Enteral nutrient intake level determines intestinal protein synthesis and accretion rates in neonatal pigs. Am. J. Physiol. Gastrointest. Liver Physiol..

[40] Takarada T., Nakamichi N., Nakazato R., Kakuda T., Kokubo H., Ikeno S., Nakamura S., Kuramoto N., Hinoi E., Yoneda Y. (2016). Possible activation by the green tea amino acid theanine of mammalian target of rapamycin signaling in undifferentiated neural progenitor cells in vitro. Biochem. Biophys. Rep..

[41] Wang L., Brennan M., Li S., Zhao H., Lange K.W., Brennan C. (2022). How does the tea L-theanine buffer stress and anxiety. Food Sci. Hum. Well..

[42] Dai Z., Li X., Xi P., Zhang J., Wu G., Zhu W. (2013). L-Glutamine regulates amino acid utilization by intestinal bacteria. Amino Acids.

[43] Matthews D.E., Marano M.A., Campbell R.G. (1993). Splanchnic bed utilization of glutamine and glutamic acid in humans. Am. J. Physiol. Endocrinol. Metab..

[44] Vanhove K., Derveaux E., Graulus G.J., Mesotten L., Thomeer M., Noben J.P., Adriaensens P. (2019). Glutamine addiction and therapeutic strategies in lung cancer. Int. J. Mol. Sci..

[45] Yokogoshi H., Kobayashi M. (1998). Hypotensive effect of γ-glutamylmethylamide in spontaneously hypertensive rats. Life Sci..

[46] Bröer S., Bröer A. (2017). Amino acid homeostasis and signalling in mammalian cells and organisms. Biochem. J..

[47] Sugiyama T., Sadzuka Y. (2003). Theanine and glutamate transporter inhibitors enhance the antitumor efficacy of chemotherapeutic agents. Biochim. Biophys. Acta.

[48] Nicholson J.K., Lindon J.C. (2008). Metabonomics. Nature.

[49] Cui Y., Han J., Ren J., Chen H., Xu B., Song N., Li H., Liang A., Shen G. (2019). Untargeted LC-MS-based metabonomics revealed that aristolochic acid I induces testicular toxicity by inhibiting amino acids metabolism, glucose metabolism, β-oxidation of fatty acids and the TCA cycle in male mice. Toxicol. Appl. Pharmacol..

