# Supplementary material for: L-Theanine Regulates the Abundance of Amino Acid Transporters in Mice Duodenum and Jejunum via the mTOR Signaling Pathway
Source: Nutrients. 2022 Dec 28;15(1):142. doi: 10.3390/nu15010142 (PMC9824403; doi:10.3390/nu15010142)
Supplement: Supplementary file 1 [file nutrients-15-00142-s001.zip › nutrients-2115089-supplementary.pdf]

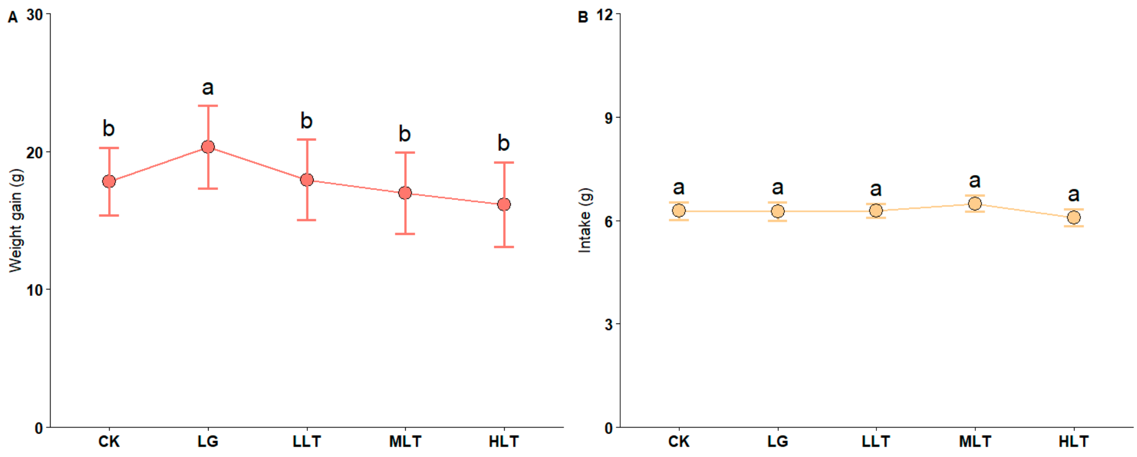

Figure S1. Effects of L-theanine on (A) weight and (B) feed intake of mice. CK (control), HLT (LTA-400 mg•kg^{-1}•d^{-1}), MLT (LTA-300 mg•kg^{-1}•d^{-1}), LLT (LTA-100 mg•kg^{-1}•d^{-1}), and LG (L-glutamine-300 mg•kg^{-1}•d^{-1}) groups. Values are presented as means ± S.D. n = 10/group. Different superscript letters in the same figure indicate statistically significant differences(P<0.05).

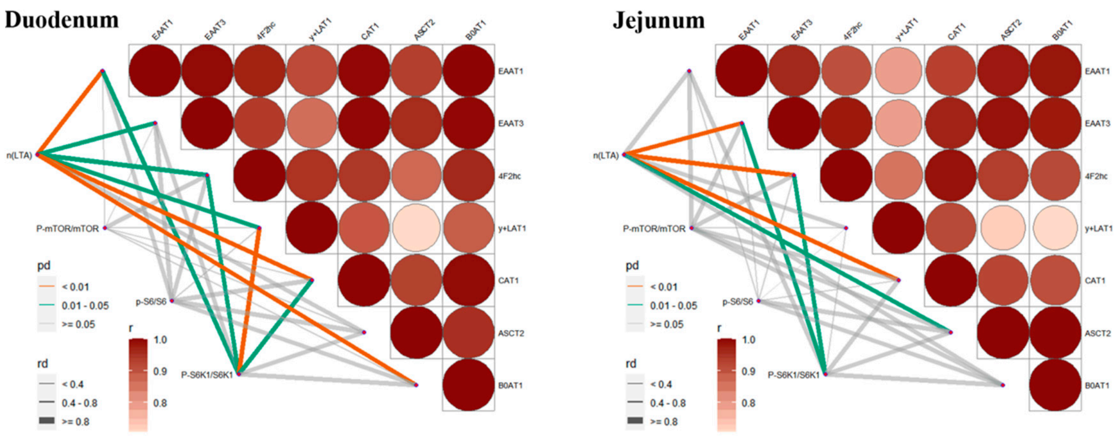

Figure S2. Correlation between the AATs protein expression and n(LTA), mTOR, S6K1, and S6. The size of the circle and the shade of the color represent the size of the correlation coefficient. pd = P-value, rd = correlation coefficient.
